# Response to a novel, weight self‐awareness plan used in a multi‐component lifestyle intervention programme to reduce breast cancer risk factors in older women—Secondary analysis from the ActWELL trial

**DOI:** 10.1111/jhn.13062

**Published:** 2022-08-02

**Authors:** Suzanne M. M. Zaremba, Martine Stead, Jennifer McKell, Ronan E. O'Carroll, Nanette Mutrie, Shaun Treweek, Annie S. Anderson, the ActWELL team

**Affiliations:** ^1^ Division of Population Health & Genomics, Ninewells Hospital & Medical School University of Dundee Dundee UK; ^2^ Faculty of Health Sciences and Sport, Institute for Social Marketing and Health University of Stirling Stirling UK; ^3^ Division of Psychology, School of Natural Sciences University of Stirling Stirling UK; ^4^ Physical Activity for Health Research Centre University of Edinburgh Edinburgh UK; ^5^ Health Services Research Unit University of Aberdeen Aberdeen UK

**Keywords:** breast‐cancer, self‐monitoring, weight

## Abstract

**Background:**

The ActWELL randomised controlled trial assessed the effectiveness of a weight management programme delivered by volunteer lifestyle coaches (LCs) in women attending breast clinics. The intervention focused on caloric intake and physical activity, utilising behavioural change techniques including a weight awareness plan (WAP). The current work is a secondary analysis of the ActWELL data and aims to examine the response to the weight self‐awareness plan (used as part of the intervention programme).

**Methods:**

The LCs invited participants (*n* = 279) to undertake an implementation intention discussion to formulate a self‐weighing (SW) plan. Bodyweight scales were offered, and recording books provided. The physical activity component of the intervention focused on a walking plan assessed by accelerometers. The LCs contacted participants by telephone monthly and provided personalised feedback. Mann–Whitney tests and chi‐squared analysis were used to examine the effect of SW on weight change. A qualitative evaluation utilising semi‐structured interviews was also undertaken.

**Results:**

Most participants (96.4%) agreed to set a weekly SW goal and 76 (27%) requested scales. At 12 months, 226 (81%) returned for follow up. The median (interquartile range) weight change for those who self‐ reported at least one weight (*n* = 211) was −2.3 kg (−5.0 to 0.0) compared to −1.2 kg (−5.0 to 0.03) in those who did not (*n* = 14). Participants who reported weights on more than eight occasions (39%) were significantly more likely (*p* = 0.012) to achieve 5% weight loss compared to those who weighed less often. Low numbers of accelerometers were returned that did not allow for significance testing. Qualitative data (*n* = 24) indicated that many participants found the WAP helpful and motivating.

**Conclusions:**

Greater adherence to the WAP initiated by volunteer coaches is associated with achieving 5% weight loss.

## Key points

- Volunteer coaches successfully supported women to lose weight in the ActWELL trial.
- Secondary analysis demonstrated that adherence to a weight awareness programme was associated with achieving 5% weight loss.

## INTRODUCTION

Excess body fat is known to be associated with at least 13 cancer types, many of which are diagnosed in older years,^1^ with the greatest burden occurring in women as a result of the association of obesity with post‐menopausal breast, endometrial and ovarian neoplasms.^2^ However, there is growing evidence to suggest that weight management (avoidance of weight gain, intentional weight loss in individuals with overweight/obesity and maintenance of weight loss) is associated with decreased cancer incidence.^3^, ^4^, ^5^ In Scotland, the breast cancer community is currently exploring opportunities to support weight management programmes for breast cancer risk reduction, utilising a community asset approach working with existing National Health Service (NHS) Breast screening sites.^6^, ^7^

The ActWELL randomised controlled trial (RCT) was designed to assess the effectiveness of a volunteer delivered weight management programme for women with a body mass index (BMI) > 25 kg m^–2^ attending four NHS Scotland Breast Screening clinics.^8^ Primary outcomes were changes in measured body weight (kg) assessed by research nurses and physical activity (step count) assessed by accelerometers between intervention and comparison groups at 12 months.^8^

The intervention was based on the COM‐B model of behaviour change.^9^ This approach was used to increase capability for lifestyle change (e.g., via a volunteer coach delivered personalised programme and providing digital skills to those who sought them), greater opportunities for being physically active (via a pedometer‐walking programme and local leisure centre use) and improve motivation for weight management (by raising awareness of lifestyle and breast cancer risk reduction within screening, and the use of goal‐setting, action and coping plans). The development of the intervention programme was based on a feasibility trial.^7^ The combination of approaches used was influenced by participant acceptability data from the feasibility study with notable concerns over programme duration, coach contact time, content and use of behaviour change techniques and communications generally (e.g., science and evidence, non‐judgemental approaches and avoiding guilt). In addition, the need for emphasis on support rather than education became apparent. These findings are detailed elsewhere.^10^ The final programme focussed on altering both caloric intake and physical activity, utilising a range of behavioural change techniques.^11^ The programme was delivered by trained volunteer lifestyle coaches (LCs) (managed by the charity Breast Cancer Now) via two (one‐to‐one) face‐to‐face visits in local leisure centres and up to nine telephone calls over a 12‐month period. The use of volunteers for supporting weight management in this context involved considerable emphasis on self‐awareness of body weight (self‐monitoring, reporting and feedback) through the use of a weight awareness plan (WAP). Reporting of body weight and feedback was the focus of all nine telephone calls. The trial results demonstrated a clinically relevant reduction in body weight in women randomised to the intervention arm who were more than twice as likely to achieve 5% loss in body weight (odds ratio = 2.20; 95% confidence interval = 1.4–3.4, *p* = 0.0005)^12^ than the comparison group. The major focus of the second primary outcome (increase step count) aimed to assess the impact of personalised, pedometer‐based walking plans with discussions on progress being discussed at monthly telephone calls. However, participants were not invited to provide specific pedometer reports (to reduce participant and coach burden). No important differences were detected in step counts between the groups at 12 months follow up.^12^

The importance of self‐monitoring in behaviour change programmes has been widely described,^13^, ^14^ and has been demonstrated to be linked with greater success in diet and physical activity interventions, in line with the principles of control theory.^15^, ^16^ Systematic review level evidence^17^, ^18^ has demonstrated that regular self‐weighing (SW) has been consistently associated with weight loss, and Shieh *et al*.^19^ reported that higher SW frequency is associated with better weight outcomes and achieving 5% weight loss. Current NICE guidelines (2014)^20^ recommend that adults include self‐monitoring of behaviour and progress in behavioural interventions for weight management.

The current work employs a novel approach in that volunteer (lay) coaches provide telephone delivered, personalised, support and feedback on self‐management of weight. This approach builds more generally on using a community assets approach to complement NHS services.

The aim of the current work was to examine the response to the weight self‐ awareness plan (used as part of the ActWELL multi‐component weight management intervention programme) among older women with excess weight who participated in the RCT. Specifically, we examined how far participants engaged with the plan, what socio‐demographic factors were associated with greatest participation in SW, the relationship between weight awareness and weight management, and participants' views on monitoring body weight.

## METHODS

The ActWELL trial recruited 560 women, aged 50–70 years with BMI > 25 kg m^–2^,^12^ via invitation cards which were provided at routine breast screening clinics. Respondents who completed the cards were contacted by telephone on a ‘first come, first served’ basis. Those who met eligibility criteria on a telephone screening call were then invited to attend their local research centre to provide informed consent and commence baseline measures prior to randomisation using the web‐based TRuST system developed and managed by the Tayside Clinical Trials Unit.^10^

### Initiating weight loss awareness procedures (intervention group only)

All ActWELL trial participants (*n* = 560) had their body height and weight measured at baseline and 12 months by research nurses at clinical research centres. For intervention participants volunteer LCs showed intervention participants (*n* = 279) how to assess their BMI (kg m^–2^) (based on measured height and weight, which were recorded in research centres and made available to LCs), aiming to identify participant weight category (overweight or obese). Weight loss goals (in kg) were then set to attain a reduction in 7% of body weight over the 12‐month intervention period. In addition, it was noted that if weight loss targets were achieved, guidance for weight loss maintenance would be provided. The control group received no advice regarding weight measurement. Full details of recruitment, randomisation, analysis and results of the ActWELL trial are presented elsewhere.^12^

### WAP (intervention group only)

The WAP was also introduced to participants at the first intervention session. To help support weekly SW procedures, LCs invited intervention participants to undertake an implementation intention^21^ discussion. Participants were asked to identify where and when they would weigh themselves, who would support them in their task (action plans), potential barriers that might arise and how these could be addressed (coping plans). Guidance was provided on weighing in a consistent manner (day, time, place) thus helping to establish a routine/habit and to minimise recording on within‐day weight fluctuations. Digital bodyweight scales were offered and record books for recording weekly weights were provided. Participants were then advised that LCs would contact them by telephone at monthly intervals and provide feedback and discussion on all weekly weight recordings, weight change and general progress on diet and physical activity (no pedometer collected step counts were collected). Notes on reported weight records and feedback provided were kept in coach logbooks for coaching purposes.

### Evaluation procedures

#### Quantitative measures

Background characteristics collected at research centres were obtained from the trial database. The key variables used for analysis were age, ethnicity, socio‐economic indicators (Scottish Index of Multiple Deprivation; SIMD),^22^ education (highest level) and home ownership status.

Coach logbooks were used to assess the number of participants who utilised implementation intention plans and the frequency with which participants disclosed their self‐weights to LCs. Participants were asked to record their weight following SW. The maximum number of participants' weights logged from the WAP telephone calls was nine (i.e., one per monthly telephone call). It was assumed that if a participant declined to inform their coach of their current weight and that either SW had not been undertaken or they did not wish to report the results, either way they did not comply with the WAP. To better assess differential outcomes, participants were divided into frequent or less frequent recorders by the mean number of weight records attained in the group.

#### Qualitative interviews

To assess the overall acceptability of the ActWELL programme, qualitative evaluation researchers (MS and JM) undertook telephone interviews with intervention participants which followed a semi‐structured topic guide. The topic guide was informed by the research objectives and by previous work conducted by the investigators exploring engagement with lifestyle interventions.^23^

All participants were asked if they would like to take part in these interviews after the final data collection for the RCT was complete. Six interviews per service area that participated in the trial were sought, from areas of both high and low deprivation quintiles (identified by SIMD). Twenty‐four participants of 26 approached for interview agreed to participate. Most participants (80%) were from higher SIMD quintiles 3, 4 and 5 (less deprived), which was consistent with overall response rates.

With the consent of the participant, interviews were audio‐recorded and transcribed verbatim and lasted on average a little under an hour. Participants were asked about their views on and experiences of different aspects of the trial design, delivery and content, including the WAP (SW, recording and telephone feedback). For the interview topic guide, see the Appendix

### Statistical analysis

Quantitative: Those missing either baseline or 12 months weight data were excluded. Univariate analysis was performed using demographic data and participant weight change. Chi‐squared tests were performed to examine the effect of SW frequency on weight change, BMI change and percent weight loss. Mann–Whitney test were used to compare median weight lost by participants who engaged or did not engage with the WAP. Analyses were conducted using SPSS, version 25 (IBM Corp.).

Qualitative: Interview transcripts were analysed thematically. Analysis began with MS reading a sample of six transcripts and producing a draft coding framework. JM undertook test coding of four transcripts using the draft framework. Once complete, MS and JM agreed a second, finalised coding framework which was applied to the full set of transcripts as facilitated by qualitative data analysis software (NVivo 12; QSR International). The finalised framework included 14 themes, with 12 of these including sub‐themes ranging in number from 2 to 11. Most of the themes included in the framework were deductive, such that they were informed by the topic guide, with a smaller number of inductive themes, arising from interviews. Themes identified from interviews were included if data relevant to these were mentioned by a substantial proportion of interviewees. Analytical procedures took account of the overall intervention methods including COM‐B components.^9^ Participant views on the WAP reported here were drawn from data coded under the sub‐theme: ‘Weighing self regularly’, under the main theme of ‘Monitoring and ActWELL documentation’.

## RESULTS

In total, 560 women were enrolled in the study of whon 279 were randomised to the ActWELL intervention arm. At 12 months, 226 (81%) returned for follow‐up measures of the primary outcome. One participant was excluded from the final analysis because of absence of baseline body weight; therefore, 225 participant data were utilised. For those who completed the study, most (90%) participants attended both face‐to‐face consultations and 59% completed all nine planned telephone calls.

In total, 24 women participated in semi structured interviews and useable data was available from all of these.

The mean age at baseline was 58.8 years, which reflects the screening policy of inviting women aged 50–70 years for routine mammography. Participants came from all socio‐economic groups; 16% were from SIMD 1 and 2 (highest areas of social deprivation). The majority were well educated and in paid employment. The mean ± SD BMI of the group at baseline was 31.0 ± 4.7 kg m^–2^. Intervention group participants who completed the study were similar in age and other socio‐demographic characteristics to all trial participants (Table 1).^12^

**Table 1:** Socio‐demographic characteristics of intervention participants at baseline and completion

	All (*n* = 279)	Completed (*n* = 225)	*p* value
Age (years), mean (SD)	58.8 (5.2)	59.1 (5.3)	0.549
Ethnicity, *N* (%)			0.259
White	273 (97.8)	223 (99.1)	–
Asian/Asian British/mixed	6 (2.2)	2 (0.9)	–
SIMD quintile, *N* (%)			0.916
1 (most deprived)	21 (7.5)	13 (5.8)	–
2	25 (9.0)	21 (9.3)	–
3	38 (13.6)	26 (11.6)	
4	65 (23.3)	53 (23.6)	–
5 (least deprived)	128 (45.9)	111 (49.3)	–
Unknown	2 (0.7)	1 (0.4)	–
Education, highest level, *N* (%)			0.954
Secondary	57 (20.4)	44 (19.6)	–
Other professional/technical qualification	90 (32.3)	75 (33.3)	–
University degree	132 (47.3)	106 (47.1)	–
Employment, *N* (%)			0.902
Retired	90 (32.3)	73 (32.4)	–
Unemployed	2 (0.7)	2 (0.9)	–
Employed, full‐time	91 (32.6)	63 (28)	
Employed, part‐time	71 (25.4)	63 (28)	–
Student, full‐time	2 (0.7)	2 (0.9)	–
Other	23 (8.2)	22 (9.8)	–
Home status, *N* (%)			0.323
Owner occupied	255 (91.4)	209 (92.9)	–
Rented	24 (8.6)	16 (7.1)	–

Abbreviations: SIMD, Scottish Index of Multiple Deprivation.

### Engagement with WAP

Overall initiation of goal setting to self‐weigh weekly and report weights at monthly intervals was high (Table 2). Of the participants who enrolled on the study, 76 (27%) requested a set of digital bodyweight scales. For those who completed the trial, 93.8% self‐reported their weights (at least once) to LCs. The mean ± SD number of times that participants self‐reported their weight during telephone calls with the LC was 7  ± 2.7.

**Table 2:** Frequency of participant engagement in personalised weight awareness plan

Set a goal to self‐weigh (weekly)	All (*n* = 279)	Completed trial (*n* = 225)
Yes	269 (96.4%)	218 (96.9%)
No	10 (3.6%)	7 (3.1%)
Total	279 (100%)	225 (100%)
Self‐reported weights given to coaches at monthly calls (at least once)		
Yes	248 (88.9%)	211 (93.8%)
No	31 (11.1%)	14 (6.2%)
Total	279 (100%)	225 (100%)

No differences were detected in socio‐economic markers (ethnicity, SIMD, education level, employment and home ownership status) between those who were below and above the mean frequency (*n* = 7) of SW (Table 3).

**Table 3:** Socio‐demographic characteristics of participants by frequency of reported self‐weighing (SW) (mean = 7)

	SW 0–7 times (*n* = 72)	SW 8–9 times (*n* = 153)	*p* value
Age in years, mean (SD)	58.6 (5.5)	59.2 (5.2)	0.429
Ethnicity, *N* (%)			0.584
White	71 (98.6)	152 (99.3)	–
Asian/Asian British/mixed	1 (1.4)	1 (0.7)	–
SIMD quintile, *N* (%)			0.421
1 (most deprived)	5 (6.9)	8 (5.2)	–
2	7 (9.7)	14 (9.2)	–
3	7 (9.7)	19 (12.4)	–
4	21 (29.2)	32 (20.9)	–
5 (least deprived)	31 (43.1)	80 (52.3)	–
Unknown	1 (1.4)	0 (0)	–
Education, highest level, *N* (%)			0.496
Secondary	11 (15.3)	33 (21.6)	–
Other professional/technical qualification	24 (33.3)	51 (33.3)	–
University degree	37 (51.4)	69 (45.1)	–
Employment, *N* (%)			0.076
Retired	21 (29.2)	52 (34)	–
Unemployed	0 (0)	2 (1.3)	–
Employed, full‐time	29 (40.3)	34 (22.2)	–
Employed, part‐time	17 (23.6)	46 (30.1)	–
Student, full‐time	1 (1.4)	1 (0.7)	–
Other	4 (5.5)	18 (11.8)	–
Home status, *N* (%)			0.949
Owner occupied	67 (93.1)	142 (92.8)	–
Rented	5 (6.9)	11 (7.2)	–

### Relationship between weight awareness and weight management

The median (interquartile range) weight loss (−2.3 kg; −5.0 to 0.0) for those who engaged in weight recording (*n* = 211; self‐weighed at least once) was greater than those (*n* = 14) who did not engage in any SW (−1.2 kg; −5.0 to 0.03; *p* > 0.05, not significant). Participants who self‐weighed most (8–9 times) were significantly more likely to achieve 3% (χ^2^ [d.f. = 1, *n* = 225] = 11.542, *p* = 0.001) and 5% (*X* ^2^ [d.f. = 1, *n* = 225] = 6.321, *p* = 0.012) weight loss compared to those who weighed less often (Table 4).

**Table 4:** Relationship between frequency of self‐reported weighing and personalised weight loss goals

	*n*	Met 3% WL goal	Did not meet 3% WL goal	Met 5% WL goal	Did not meet 5% WL goal
SW frequency 0–7 times	72	23	49	16	56
		(31.9%)	(68.1%)	(22.2%)	(77.8%)
SW frequency 8–9 times	153	86	67	60	93
		(56.2%)	(43.8%)	(39.2%)	(60.8%)

Abbreviation: SW, self‐weighing; WL, weight loss.

### Participants' views on weight awareness procedures

#### SW and recording

Views on the acceptability of SW varied. Many participants reported being content to weigh themselves on a regular basis, with some mentioning that they had been used to monitoring their weight regularly. Although some found the task helpful and motivating, others felt that checking their weight could be demotivating.

… keeping a wee note of your weight was quite a good wee monitor. A wee jag to keep you going (Participant A, Service 1)

I wasn't too happy when I hadn't lost anything. Even a pound or so was fine. No, it was just pack (sic) and parcel of the whole deal. It was quite good looking at the chart at the very beginning, and when you were getting to a certain stage you thought, god, how many pounds is that. It was really good … (Participant F, Service 2)

I'm not a great one for getting on the scales, because I think I tend to go by my clothes and I know if I've put on a bit or I've lost a bit or whatever, so I tend … I don't like to stand on the scales. I think that can be a bit of a demotivator, for me anyway. I know that there's other people like to stand on the scales every single day, which is ridiculous. So for me I know how I feel about myself and about my body (Participant G, Service 3)

Some participants mentioned that they did not have access to a set of weighing scales or one that was accurate and were pleased to have received a set from the LCs.

[LC's coach] gave me the use of a set of scales, so that was good, because I didn't have any scales. That was good to have accurate scales (Participant B, Service 2)

#### Telephone feedback

Participants generally welcomed the regular phone calls from LCs to discuss progress with regard to their goals (although there was an overall preference for face‐to‐face communication during the intervention). LCs were perceived as motivating and non‐judgmental, providing praise for success and encouragement when setbacks were experienced.

As I say, I had a good rapport with [LC] so I actually looked forward to them (phone calls). I was never – even if I'd put on (weight), I didn't feel, oh god, here we go. I never felt under pressure. I never felt under pressure at all with the phone calls or anything (Participant D, Service 1)

She would say oh you've lost two pounds this week, or you've lost four pounds in a fortnight. You're doing well, blah, blah, blah. So that kind of spurs you on (Participant C, Service 1)

Participants also felt that the prospect of receiving calls was helpful in motivating them to keep going with the changes they had made due to a desire to want to demonstrate progress. In this context, SW, and the associated accountability of reporting it to someone else, was often key to this process.

And it, kind of, got you motivated ‘cause you knew that she was going to phone you and you knew that you had to behave, if you like, before she phoned. ‘Cause you wanted … you desperately wanted this weight when you … when she phoned, you desperately wanted to tell her that your weight was down … I don't know if ‘checking up on you' is the right word or not, but … or being, you know … phoning you to keep an eye on you or whatever, however you want to word it, it does, sort of, give you incentive to not put that biscuit in your mouth. To make sure … you know, even if it is raining, go out for your walk or whatever (Participant C, Service 4)

It's something (weighing self) I really needed to do. It was like a little bit of incentive you know, thinking I'm going to have to write down my weight and I'm going to have to tell them and I don't want to be going in the wrong direction. So it was just a help. You just don't feel as if you're on your own with it (Participant B, Service 1)

… don't get me wrong, I have fallen off the bandwagon several times in a spectacular way but I was always aware that [LCs] would be phoning me and I would have to get back onto the bandwagon (Participant E, Service 4)

However, a few participants expressed some dissatisfaction with the phone calls, feeling that there was a lack of rapport and limited value in the contact with the coach when it was not in person. Instead, face‐to‐face contact fostered a greater feeling of accountability.

I had the phone calls and I just felt I could be saying anything to the lady over the phone, you know, like oh yes, I'm walking, I'm doing this, I'm doing that. She really had to believe what I was saying (Participant G, Service 4)

## DISCUSSION

The results indicate that the WAP used within a 12‐month, multi‐component weight management intervention was successfully initiated and supported by LCs. For women who attended both the ActWELL baseline and 12‐month measurement visits, engagement with the WAP was associated with a greater likelihood of achieving up to 5% weight loss and was acceptable to participants.

The strength of the present study includes the novel delivery of a WAP programme which was informed by the COM‐B framework and aimed to improve capability for weighing by offering scales (which a notable 27% accepted), opportunity for recording and receiving feedback, and motivation through the use of implementation intentions, action and coping planning and LC contact and support. Efforts were made to avoid potential inequality issues by offering free scales, minimising recording procedures and utilising coach‐initiated feedback. Qualitative data suggests that participants were generally satisfied with the number, frequency and timing of LC calls, which is supported by the high numbers (211 of 225; 93.8%) undertaking the WAP procedures and that these helped participants to feel accountable, receive neutral feedback and maintain motivation. However, the WAP used in the present study was not tested as a single intervention and thus it is not possible to estimate the effect size of the WAP *per se* because it is likely that other programme features (e.g., personalised advice, social support) contributed to the weight loss achieved by the intervention group.

Qualitative results suggest that not all participants appreciated SW, but it is not possible to identify whether this may have been a reason for study drop out in the 19% who failed to complete the ActWELL trial. Not knowing the 12‐month weight of these participants, and therefore being unable to include them in the present study, is a limitation. The number of participants who provided a 12‐month measurement but did no SW is small (14 of 225), which does suggest that women who did not self‐weigh may have been more prone to miss the 12‐month measurement. These 14 women lost less weight than the SW majority and, perhaps, had we had 12‐month data from all women, our confidence in this result would be stronger. As it is, uncertainty about the 12‐month weight of the 19% of women who failed to complete the trial remains and is of course a limitation and an interesting area to explore in future studies.

Our findings agree with systematic review findings by Shieh et al.^19^ in that higher SW frequency is associated with better weight outcomes and achieving 5% weight loss. Both daily and weekly SW are associated with positive weight management outcomes, but weekly approaches reduce participant burden. There is evidence to support the beneficial role of successful weight loss with higher frequencies of SW in females in their fifth and sixth decade of life when SW of body weight is the primary intervention.^24^, ^25^, ^26^ Yet, successful weight loss is likely to be achieved as part of a multi‐faceted behaviour change approach, involving not only self‐monitoring, but also strategies such as goal setting and changing beliefs and expectations. Similarly, weight monitoring is likely to be beneficial in weight loss maintenance^27^, ^28^ although it is unclear how useful this is after feedback is no longer available.

Overall, the findings of the current work suggest that the novel approach of delivering the intervention including the WAP by volunteer LCs is a useful tool in the portfolio of weight management techniques. It is not clear whether the feedback from coaches motivated adherence (and therefore continued weight loss) or whether participants who were already adhering well were more likely to report to coaches. In the current context of women attending routine breast screening clinics, the WAP offers a starting point for raising awareness of excess body weight and a prompt for weight management action. Whether self‐reporting would be an accurate record of weight change (when no independent body weight measurements are made for comparison) is unknown, although recent developments in smart scale technology could assist in increasing validity_._ ^29^, ^30^

Further work is needed to explore the long‐term usage of the WAP programme in the intervention group, factors that have influenced its continuation and continuing weight trajectories, particularly weight loss maintenance.^31^ Further work could also explore the potential of feedback on step count recording, which could help participants achieve greater physical activity (no significant difference in step count was achieved in the RCT.^12^

In conclusion, a WAP initiated by volunteer LCs as a part of a multi‐component weight loss intervention is generally acceptable to users and greater frequency of weight reporting is likely to be associated with achieving 5% weight loss.

## AUTHOR CONTRIBUTIONS

**Suzanne M. M. Zaremba**: Research concept and design, data analysis, drafting publication. **Martine Stead**: Research concept, interview design, interviewing, qualitative analysis, drafting publication. **Jennifer McKell**: Research concept, interview design, interviewing, qualitative analysis, drafting publication. **Nanette Mutrie**: Research concept and design, data collection design, guidance on analysis and interpretation of physical activity. **Ronan E. O'Carroll**: Research concept and design, guidance on psychological concepts, interpretation, drafting publication. **Shaun Treweek**: Research concept and design, data collection design, data interpretation, drafting publication. **Annie S. Anderson**: Research concept and design, data collection design, interview design, data interpretation, drafting publication.

## CONFLICTS OF INTEREST

The authors declare that there are no conflicts of interest.

## TRANSPARENCY DECLARATION

The lead author affirms that this manuscript is an honest, accurate and transparent account of the study being reported. The lead author affirms that no important aspects of the study have been omitted and that any discrepancies from the study as planned have been explained. The protocol for the ActWELL study was approved by East of Scotland Research Ethics Committee (17/ES/0073). All participants provided written informed consent for data analysis before participation.

## PEER REVIEW

The peer review history for this article is available at https://publons.com/publon/10.1111/jhn.13062.

## Appendix

ActWELL Participant Exit Interview Topic Guide

**Introduction**

Personal introduction followed by explanation of purpose of interview and focus upon experience of ActWELL study. **Emphasise researcher independence from study team and counsellors and encourage expression of candid thoughts and opinions**. Check for any questions from participant. Introduce voice recorder and check that participant is comfortable with use of voice recorder.

1. **Background lead in – as appropriate**
  - Age; place of residence; family circumstances
  - Current general health
2. **Introduction to study (and expression of interest)**
  - How first became aware of ActWELL study? recall of discussion with mammography staff – were they encouraging or neutral?
  - Initial thoughts/responses to the study
  - Reasons for expressing interest in participation
  - Recall of receiving study information and any letter of endorsement from lead doctors (local area)
3. **Study team contact; deciding to take part and recruitment to the study**
  - Recall of first contact with study team a week after clinic appointment (by email or by phone?)
    - Impressions of researcher
    - Understanding of process proposed
    - Any queries/concerns
    - Thoughts on collection of all measurement data including height and weight data – was it too much
    - Was the follow up visit helpful/acceptable
  - Recall of receiving full study information sheet and sufficiency and clarity of information
  - Thoughts on provision on opt‐out slip and prospective appointment
  - Thinking about taking part in study and discussion with others: friends/family; professionals/study team (opportunity to ask questions?)
  - Factors encouraging participation; for example, a desire to help; to feel fitter/healthier
  - Factors discouraging participation; for example, time and practicalities involved
  - Final reasons balance of decisions for taking part
  - Previous experience of lifestyle change
  - Attending study centre
    - Ease/cost of attendance with relative/friend
    - Duration/timeliness of appointment
    - Coverage of issues/queries answered
    - Thoughts on collection of baseline measurements by staff
    - Perspective of staff conducting appointment
    - Thoughts on inclusion of formal consent procedure at this stage
    - Thoughts on the randomisation process
  - Expectations and understanding of what the study would entail: any remaining concerns or anxieties
4. **Face to face appointment with lifestyle coach (× 2 in leisure centre/community setting)**
  - Any expectations prior to attending the appointments
  - Recall of appointment including when it took place, with whom and duration (sufficient?)
  - Thoughts on approach and manner of coach (check what language they use to describe the coaches title/role)
  - How they felt about the idea of changing diet and activity
  - Any goals set
5. **Phone‐calls (up to 9 over period of 12 months)**
  - How well did conversations with the counsellor work on the phone – comparison with face‐to‐face contact
  - Any issues around fixing up times for calls, privacy, convenience and so on
  - Probe recall of what was discussed in each call, what advice the counsellor gave; any changes in the calls as months progressed (e.g., more cursory/more in‐depth)
  - Progress towards meeting goals, sticking to plans, any setbacks and impact and so on
6. **Info and advice given (also use of pedometer)**
  - Recall of information, tips and strategies suggested by the counsellor concerning:
    - Diet: substitution of foods (e.g., brown rice for white), portion size and control, calorie control, cooking methods, snacks and so on
    - Weight loss: target amount, frequency of weighing self
    - Physical activity: steps or other targets, frequency of activity, type of activity, substitution/incorporation into everyday life (e.g., walking the longer way back from the shops) and so on
  - Usefulness of pedometer and associated walking programme
  - Usefulness of ActWELL information pack
7. **General aspects of participation (if not already covered above)**
  - Experience of monitoring
    - Weighing self
    - Collection of follow up measurements
  - Overall understanding of the programme and objectives
    - Understanding purpose of different aspects: exercise, weight loss, diet changes
    - Goal setting – how decided, perspectives including understanding of expectations
  - Changes made in terms of exercise, weight and diet
  - Any barriers to making changes
  - Any facilitators to making changes
  - Ability and success in maintaining changes
  - Satisfaction or otherwise with progress made
  - What if anything they would do differently if participating in the programme again
  - Other

8. **Social support**
  - Perceived response (encouragement, support and so on) from
    - Family, especially those living in same home
    - Friends
    - Others; for example, professionals, such as GPs
  - Wider community context (if not already covered above): how easy is it to make changes to diet in terms of what's available in the local food shops/the sorts of food you can afford; how easy is it to do more physical activity living here/given your lifestyle and ability?

9. **Overview**
  Whether breast screening clinics are acceptable/feasible setting for recruitment into a lifestyle intervention
  Initial expectations match experience of taking part?
  Advantages
  Disadvantages
  What parts liked least/liked most
  Any parts of the programme that could be improved
  Whether would recommend or discourage others to take part in programme
  Other
